# Severe Burn Injury Significantly Alters the Gene Expression and m6A Methylation Tagging of mRNAs and lncRNAs in Human Skin

**DOI:** 10.3390/jpm13010150

**Published:** 2023-01-12

**Authors:** Yanqin Ran, Zhuoxian Yan, Mitao Huang, Situo Zhou, Fangqin Wu, Mengna Wang, Sifan Yang, Pihong Zhang, Xiaoyuan Huang, Bimei Jiang, Pengfei Liang

**Affiliations:** 1 Department of Burns and Plastic Surgery, Xiangya Hospital, Central South University, Changsha 410008, China; 2 Department of Pathophysiology, Xiangya School of Medicine, Central South University, Changsha 410008, China

**Keywords:** human skin, m6A, wound healing, epigenetics, microarray, burn injury

## Abstract

N6-methyladenosine (m6A) modulates RNA metabolism and functions in cell differentiation, tissue development, and immune response. After acute burns, skin wounds are highly susceptible to infection and poor healing. However, our understanding of the effect of burn injuries on m6A methylation and their potential mechanism is still limited. Human m6A-mRNA&lncRNA Epitranscriptomic microarray was used to obtain comprehensive mRNA and lncRNA transcriptome m6A profiling and gene expression patterns after burn injuries in human skin tissue. Bioinformatic and functional analyses were conducted to find molecular functions. Microarray profiling showed that 65 mRNAs and 39 lncRNAs were significantly hypermethylated; 5492 mRNAs and 754 lncRNAs were significantly hypomethylated. Notably, 3989 hypomethylated mRNAs were down-expressed and inhibited many wound healing biological processes and pathways including in the protein catabolic process and supramolecular fiber organization pathway; 39 hypermethylated mRNAs were up-expressed and influenced the cell surface receptor signaling pathway and inflammatory response. Moreover, we validated that m6A regulators (METTL14, METTL16, ALKBH5, FMR1, and HNRNPC) were significantly downregulated after burn injury which may be responsible for the alteration of m6A modification and gene expression. In summary, we found that homeostasis in the skin was disrupted and m6A modification may be a potential mechanism affecting trauma infection and wound healing.

## 1. Introduction

The skin is the largest organ system in the body. As such, it plays a pivotal role in the protection against mechanical forces and infections, fluid imbalance, and thermal dysregulation [1]. Severe burns are the most traumatic and debilitating injuries to the skin and result in significant morbidity and mortality [2]. Deep burns alter the integrity of the skin’s sensitive innervation and affect the patient’s quality of life by impairing the perception of touch, temperature, and pain [3]. The dermis is made of a large network of collagen fibers and blood vessels that offer mechanical support and maintain the appearance of the skin, playing an important role in wound repair [4,5]. After severe large burns to the skin, the dermis usually becomes denatured and develops immune and inflammatory response dysregulation, cellular metabolic disorders, and significant morphological changes [6].

At present, an increasing number of studies have found epitranscriptomics to be essential for maintaining skin homeostasis and aiding the wound healing process [7,8,9]. Epigenetic pathways play a key role in coordinating the behavior and activity of the multitude of cell types seen during skin repair [10]. Given the complexity of the wound healing process and the requirement of stringent regulation, epigenetic regulation, including histone modifications and DNA methylation, is highly likely to play a role [11,12,13,14,15]. RNA methylation modifications play a key role in coordinating the behavior and activity of the multitude of cell types seen during skin wound repair [16,17]. 

N6-methyladenosine (m6A) is one of the most abundant post-transcriptional modifications found in eukaryotic mRNA. M6A is highly enriched in the 5′ untranslated regions (UTR), coding sequence (CDS), stop codon, and 3′ UTR [18,19]. In the nucleus, m6A modification of the mRNA is dynamically and reversibly regulated by methyltransferases (“writers”), demethylases (“erasers”), and binding proteins (“readers”) [20]. mRNAs can be methylated and demethylated by writers and erasers, whose dysregulation is often associated with disease by paradoxically increasing or removing m6A from RNA transcripts with critical biological functions [21,22]. Writers and erasers may target different transcriptomes in different cell types and different biological processes. Erasers and different readers may also regulate or recognize m6A in specific regions of transcripts, leading to different functional outcomes [23]. m6A modifications affect gene expression by regulating mRNA stability, splicing, translation, and other metabolic processes [24]. m6A RNA methylation regulates oncogene expression, cancer stem/initiating cell pluripotency, cell differentiation, cell proliferation, migration, and angiogenesis [25]. 

Studies have found that m6A modification plays a role in various skin wounds by regulating the proliferation and migration of keratinocytes [26], differentiation of stem cells [27], the onset of apoptosis [28], and regeneration of lymphatic vessels [29]. However, our understanding of the effect of burn injuries on skin m6A methylation and the underlying pathophysiological responses after burn injuries is still limited.

In this study, we aim to characterize the molecular changes, functional alterations, and m6A modification variations in the skin triggered by severe burns. Using human m6A-mRNA&lncRNA Epitranscriptomic microarray, we identified the differentially m6A methylated mRNAs and lncRNAs and revealed the potential relationship between RNA methylation modification and differentially methylated and expressed genes (DMEGs). Gene Ontology (GO) and pathway analysis were performed to better understand the differentially expressed and methylated mRNAs.

## 2. Materials and Methods

### 2.1. Collection of Normal and Burn-Injured Skin

Burned skin and normal uninjured skin tissue from five patients were included in this study. In this study, the use of burn-injured skin was approved by the Research Ethics Board at Xiangya Hospital (NO. 2019030421), and all required consent was obtained from patients. A total of five pairs of burn-injured skin samples was collected from five men with second and third-degree burns who were admitted to the hospital within 24 h of burn injury. Samples were collected during tangential excision of eschar and large split-thickness autografts with preserved dermis on days 1–2 after the burn injury. Normal skin was obtained from the patient after the skin grafting procedure. We incubated the normal skin overnight at 4 °C for 12 h in Dispase II (0.4 mg/mL, Sigma Aldrich, St. Cat. D4693, Burlington, MA, USA) to obtain normal dermal tissue. After we separated the epidermis from the normal skin using fine forceps the next day, the remaining normal dermis was then three times washed with phosphate-buffered saline (PBS) and stored in liquid nitrogen for further research. Burn-injured skin identification: scorched scabs and yellow necrotic tissue were cleared and then a layer of the white dermis with a few scattered tiny blebs was seen, confirming the white dermis as burn-injured skin for further experiments.

### 2.2. Methylated RNA Immunoprecipitation

Total RNA isolated from five pairs of burn-injured skin and normal skin was quantified using the NanoDrop ND-1000. RNAs were incubated and immunoprecipitated (at 4 °C for 2 h) with an anti-N6-methyadenosine (m6A) antibody (202003, Synaptic System, Goettingen, Germany). The RNA with m6A modification was eluted at 50 °C for 1 h, and the RNA without m6A modification was recovered from the supernatant. Then, the RNA with m6A modification was extracted by acid phenol-chloroform and the RNA without m6A modification was extracted by ethanol precipitation.

### 2.3. Microarray Hybridization

The immunoprecipitated “IP” fraction contained enriched m6A methylated RNAs, and the supernatant “Sup” fraction contained unmodified RNAs. “IP” and “Sup” RNAs were amplified as cRNAs and labeled with Cy5 and Cy3 fluorescent dye, respectively, using the Arraystar Super RNA Labeling Kit. Cy3 and Cy5 labeled cRNAs were combined and hybridized to Arraystar Human mRNA&lncRNA Epitranscriptomic Arrays (8 × 60 K, Arraystar) that contained 27,413 mRNAs and 5553 lncRNAs degenerate probes at 65 °C for 17 h in an Agilent Hybridization Oven. After washing the slides, the arrays were scanned in two-color channels by an Agilent Scanner G2505C.

### 2.4. Microarray Data Analysis

The acquired array images were analyzed by Agilent Feature Extraction software (version 11.0.1.1). After normalizing raw intensities of IP (immunoprecipitated, Cy5-labelled) and Sup (supernatant, Cy3-labelled) with an average of log2-scaled Spike-in RNA intensities, the fold change and *p* values were calculated for each transcript between burns-injured skin and normal skin. The m6A methylation level was calculated for the m6A methylation amount based on the IP (Cy5-labelled) normalized intensities. The mRNA expression level was calculated for the IP (Cy5-labelled) and Sup (Cy3-labelled) normalized intensities. A cutoff of 4-fold (*p* < 0.05) was used to identify the differentially m6A methylated RNAs in burn-injured skin compared to normal skin.

### 2.5. Quantitative Real-Time Polymerase Chain Reaction (qRT-PCR)

The mRNA levels of m6A regulators (writer: METTL3, METTL14, WTAP, METTL16; eraser: ALKBH5, FTO; reader: FMR1, LRPPRC, and HNRNPC) were estimated by real-time polymerase chain reaction analysis with the SYBR-green method using gene-specific primers. GAPDH was used as an endogenous control. The primers and sequences used in this study are presented in Table 1.

### 2.6. Gene Ontology (GO) and Kyoto Encyclopedia of Genes and Genomes (KEGG) Analyses

GO analysis was performed to explore the potential function of the differentially expressed genes (DEGs), differentially methylated genes (DMGs), and differentially methylated and expressed genes (DMEGs) in the burn-injured skin when compared with normal skin to describe the potential biological process (BP). Pathway analysis for DEGs, DMGs, and DMEGs was used to identify the pathways according to KEGG (http://www.genome.jp/kegg/, accessed on 1 June 2022). Enrichment scoring, which is equal to −log10 (*p* value), was used to measure pathway significance and specificity. 

### 2.7. Protein–Protein Interaction Network

The STRING database (https://www.string-db.org/, accessed on 5 June 2022), which has detailed information about protein interactions, was used to import the DMEGs, and from there, the link between DMEGs was determined. The data were imported into the Cytoscape program (v3.9.0, National Resource for Network Biology, https://cytoscape.org/, accessed on 5 June 2022), which then built the protein–protein interaction (PPI) network, and then, the network was analyzed by Network Analyzer. The DEGs with interactions with combined scores greater than 0.4 were selected to construct a protein-protein interaction network diagram 2.

### 2.8. Statistical Analysis

All data from three or more independent experiments are presented as mean ± SD. Student’s *t*-test and one-way analysis of variance were used to analyze significant differences using SPSS 16.0 software. A value of *p* < 0.05 represented a statistically significant difference. Using the Benjamini and Hochberg method, *p*-values were adjusted for multiple testing to control the false discovery rate (FDR). Differentially methylated mRNAs and lncRNAs were identified using stringent-filtering criteria (|fold change| ≥ 2, *p* < 0.05). Heatmap and volcano plots were generated with the “heat-map” and “ggplot2” packages in R (version 4.1.0). Bioinformatic analysis was performed using the OmicStudio tools at https://www.omicstudio.cn/tool, accessed on 1 June 2022.

## 3. Results

### 3.1. Identification of Burn Injury-Induced Changes in mRNA Expression Profiling and Function Enrichment

**Figure 1:**
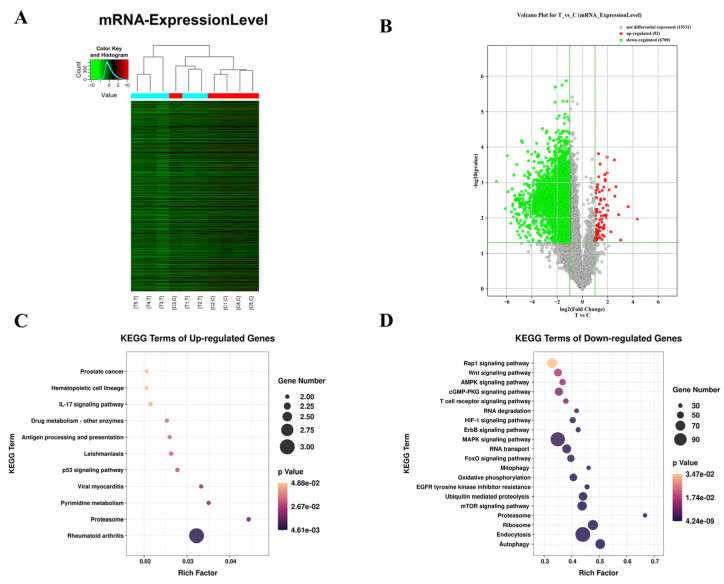
Changes in the expression level of mRNA transcripts in burn-injured skin compared to normal skin tissues. (**A**) Hierarchical clustering analysis of differentially expressed transcripts between burn-injured skin and normal skin tissue. At the bottom of the image, T and C represented burn-injured skin samples and normal skin samples, respectively. Red represented a significant increase and green represented a significant decrease. (**B**) Volcano plot analysis of differently expressed mRNA transcripts of genes between the two groups. Red represented a significant increase and green represented a significant decrease (|fold change| ≥ 2, *p* < 0.05). (**C**,**D**) The KEGG pathway enrichments of up- and down-regulated genes. The size of the dot represented the number of genes enriched to that pathway; the color of the dot represented the size of the *p* value enriched to that pathway; the horizontal coordinate represented the ratio of the proportion of differentially expressed proteins annotated to that pathway to the proportion of proteins annotated to a pathway in that species. The larger the enrichment factor, the more reliable the enrichment significance of the differential protein in that pathway.

To investigate the alteration of gene expression at the transcriptomics induced by burn injuries, the level of mRNA expression was detected in the burn-injured skin and normal skin by analyzing the Cy3 and Cy5 fluorescent dye intensity of mRNAs in our microarray. Hierarchical clustering analysis of differentially expressed transcripts was performed between the burn-injured skin and normal skin tissue (Figure 1A). A total of 6770 mRNA transcripts were differentially expressed (|fold change| ≥ 2, *p* < 0.05; *n* = 5/group). 6700 mRNAs were significantly downregulated in the burn-injured skin compared to normal skin tissue, and 70 mRNAs were significantly upregulated (Figure 1B). Moreover, KEGG analyses were used to explore the potential function of the differentially up- and down-expressed mRNAs. Interestingly, we found that the up-expressed genes including CXCL5, RRM2, MMP1, HLA-DRB5, and PSME3 were mainly enriched in the p53 signaling pathway, IL-17 signaling pathway, and Antigen processing and presentation (Figure 1C). The down-expressed genes were closely related to the autophagy pathway, mTOR signaling pathway, proteasome, and MAPK (mitogen-activated protein kinase) signaling (Figure 1D), which play critical roles in promoting wound healing. By differential expression analysis at the mRNA transcriptome level, we found that the expression of most genes was repressed after thermal injury, which may lead to difficult wound healing.

### 3.2. Expression Alteration of m6A Regulators during the Recovery of the Burn-Injured Skin

**Figure 2:**
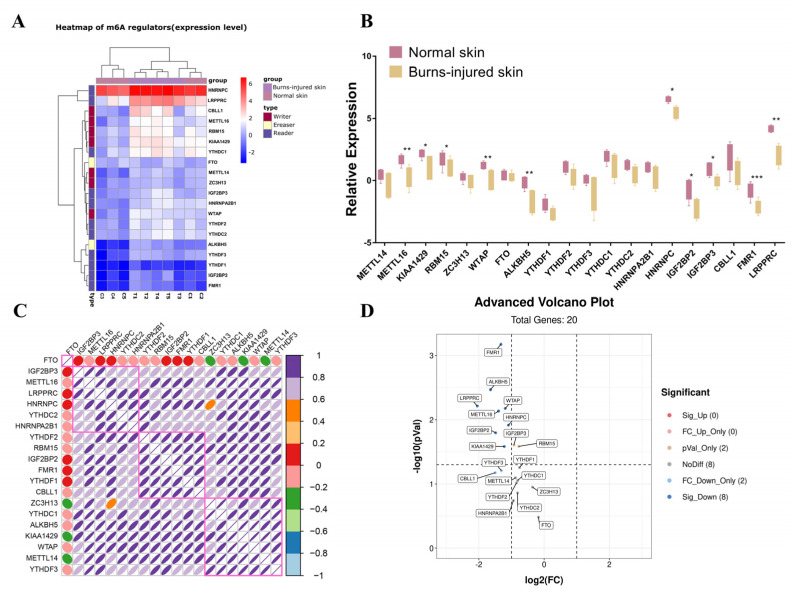
Expression alterations of m6A regulators after burn injury. (**A**) Hierarchical clustering analysis of differentially expressed m6A regulators between normal skin and burn-injured skin. At the bottom of the image, T and C represented burn-injured skin samples and normal skin samples, respectively. Red represented a significant increase and green represented a significant decrease. (**B**) Box plot analysis of transcriptome expression status of 20 m6A regulators between normal tissues and burn-injured skin. The lines in the boxes represented medians. The upper and lower ends of the boxes represented the interquartile range of values. Asterisks represented statistical *p*-values (* *p* < 0.05; ** *p* < 0.01; *** *p* < 0.001). (**C**) Correlations between m6A regulators using Spearman analysis. The area of the shuttle represented the size of the statistical *p*-value. The smaller the area, the smaller the *p*-value, indicating a larger statistical difference. The different colors represented the magnitude of the correlation. (**D**) Scatter plot shows the summary of expression changing information of differentially expressed m6A regulators between normal skin and burn-injured skin.

To investigate whether m6A methylation status was changed and the potential function in the burn-injured skin, we also evaluated the mRNA expression level of the m6A regulators in the microarray. The expression of METTL3, ZFP217, RBM15B, IGF2BP1, and ELAVL1 was eliminated because of low signal in the microarray. The hierarchical clustering heatmap and box plot demonstrated that FMR1, ALKBH5, LRPPRC, WTAP, METTL16, HNRNPC, IGF2BP2, IGF2BP3, RBM15, and KIAA1429 was significantly downregulated in the burn-injured skin, compared with normal skin (*p* < 0.05) (Figure 2A,B). The expression relationship among 20 m6A regulators was compared by Spearman correlation analysis in the 10 samples between the burn-injured skin and normal skin (Figure 2C). We demonstrated that there are three clusters: YTHDF2, RBM15, IGF2BP2, FMR1, YTHDF1, CBLL1; ZC3H13, YTHDC1; ALKBH5, KIAA1429, WTAP, METTL14, and YTHDF3 which have a significant relationship in the burn-injured skin, indicating that they function together. Differentially expressed analysis identified 20 expressions altered m6A regulators, where LRPPRC had the largest fold change and FMR1 had the most statistically significant change in the box plot (Figure 2D). 

**Figure 3:**
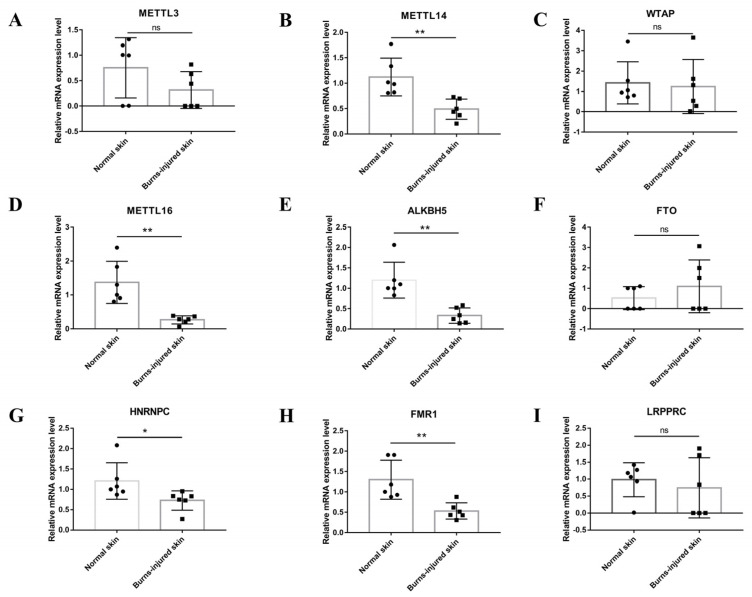
Validations of the mRNA expression levels of m6A regulators in burn-injured skin and normal skin. (**A**–**I**), RT-PCR of the nine m6A regulators (writer: METTL3, METTL14, WTAP, METTL16; eraser: ALKBH5, FTO; writer: METTL3, METTL14, WTAP, METTL16; eraser: ALKBH5, FTO; reader: FMR1, LRPPRC, and HNRNPC) based on *p*-value in burn-injured skin and normal skin (*n* = 6 per group). Data are shown as mean ± SD, and the differences were determined by Student’s *t*-test. * *p* < 0.05, ** *p* < 0.01, and ns presents no significance, compared with the control group.

We selected nine m6A regulators (writer: METTL3, METTL14, WTAP, METTL16; eraser: ALKBH5, FTO; reader: FMR1, LRPPRC, and HNRNPC) for PCR validation based on *p* values (Figure 3A–F). Our results showed that the expression levels of METTL14, METTL16, ALKBH, FMR1, and HNRNPC were consistent with the microarray results. 

### 3.3. Significant Alteration of m6A-Modified mRNAs and lncRNAs

**Figure 4:**
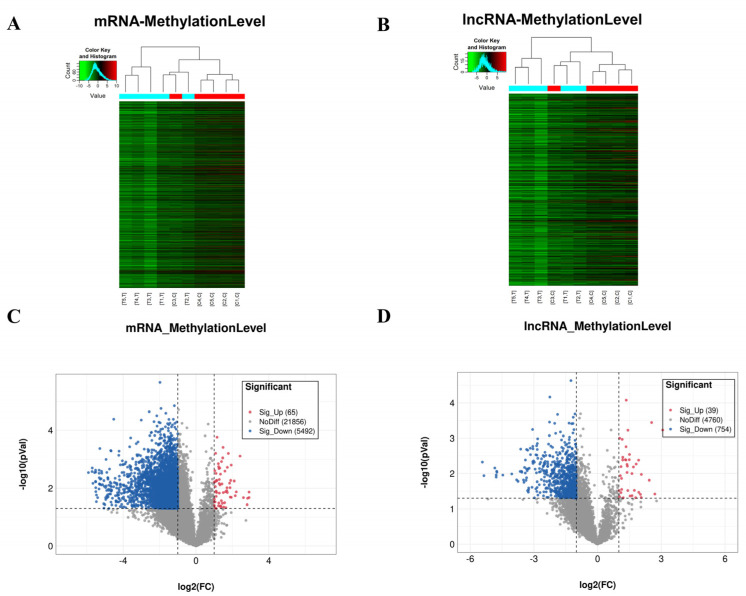
Changes in m6A modification profiles of mRNA and lncRNA transcripts after burn injury compared to normal skin. (**A**,**B**) Hierarchical clustering analysis of differentially m6A methylated transcripts between burn-injured skin and normal skin. (**C**,**D**) Volcano plot analysis of different methylated mRNA and lncRNA transcripts. Red represented a significant increase and blue represented a significant decrease (|fold change| ≥ 2, *p <* 0.05).

Based on the altered expression of m6A and the interaction relationship, we profiled the immunoprecipitated m6A methylated mRNAs and lncRNAs (27413 mRNAs and 5553 lncRNAs) isolated from normal skin and burn-injured skin and labeled them with Cy5 fluorescent dye to determine the m6A changes and the effect of burn injuries on transcript-specific m6A changes. Depending on the similarities in the levels of m6A methylation on mRNAs and lncRNAs, hierarchical clustering was used to categorize the samples and identify their linkages between the two groups (Figure 4A,B). Volcano plot analysis showed that 65 mRNAs and 39 lncRNAs were significantly hypermethylated, while 5492 mRNAs and 754 lncRNAs were significantly hypomethylated in the burn-injured skin compared with normal skin (|fold change| ≥ 2, *p* < 0.05; *n* = 5/group) (Figure 4C,D).

These data demonstrated that burn-injured skin and normal skin display distinct m6A-modified mRNA and lncRNA patterns. m6A homeostasis is achieved through a coordinated activity of the m6A methylase complex and demethylases. After severe burn injury, the expression of m6A writers and erasers was down-regulated, which may induce the m6A alteration of mRNAs. For the hypermethylated mRNAs and lncRNAs, we believe that m6A erasers (ALKBH5) may play the key role in m6A modification; and for the hypomethylated mRNAs and lncRNAs, the m6A writers (METTL14 and METTL16) may critical. 

### 3.4. Functional Analysis of Differentially m6A-Modified Genes in the Two Groups

**Figure 5:**
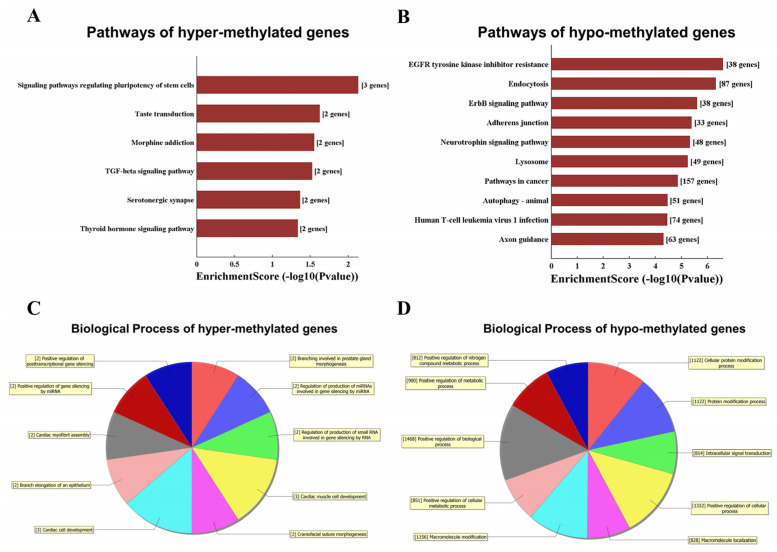
GO analyses and KEGG pathway analyses of differentially m6A modified genes. (**A**,**B**) KEGG pathway analysis results of hyper- and hypo-methylated m6A modified mRNA-associated genes. (**C**,**D**) Gene Ontology functional enrichment analysis results of hyper- and hypo-methylated m6A modified mRNA-associated genes. The color represented the different biological process terms, and the number behind the biological process term represented the number of genes enriched by the biological process term.

We noticed that the modification level of m6A was significantly altered after burn injuries, and to explore which important pathways were affected, we conducted the KEGG pathway analysis. The hypermethylated genes were mostly enriched in signaling pathways regulating the pluripotency of stem cell pathways and TGF-beta signaling pathways between the two groups (Figure 5A). By performing enrichment analysis on hypomethylated genes, we found that the EGFR tyrosine kinase inhibitor resistance pathway, endocytosis pathway, ErbB signaling pathway, and adherens junction pathway were affected and inhibited after burn injury (Figure 5B). Furthermore, it is also crucial to analyze the biological process of differentially methylated genes. GO analysis was performed to explore the differentially methylated genes’ potential biological process (BP). In our study, the genes with a significant m6A hypermethylation level were mainly enriched for GO terms related to the regulation of production of small RNA involved in gene silencing by RNA, branch elongation of epithelium, and developmental growth involved in morphogenesis (Figure 5C). The genes enriched in the cellular protein modification process, intracellular signal transduction, and positive regulation of the cellular metabolic process were hypomethylated after burn injuries (Figure 5D).

### 3.5. Combined Analysis of Genes with Different Expression Levels and m6A Methylation Levels

**Figure 6:**
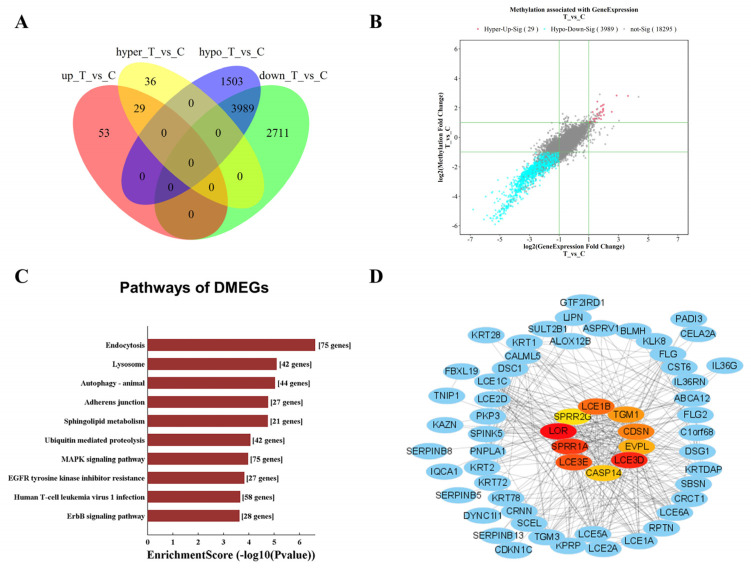
Combined analysis of genes with different transcriptome expression levels and m6A methylation levels after burn injuries compared to normal skin. (**A**) Venn diagram of differentially m6A methylated and differentially expressed genes. T and C represented burn-injured skin samples and normal skin samples. (**B**) Scatter plot analysis showed the correlation between the m6A modification level and transcriptome expression level of DMEGs. The horizontal coordinate indicated the differential ploidy of the transcriptome, and the vertical coordinate indicated the differential ploidy of m6A methylation in burn-injured skin compared to normal skin. Each dot represented a gene, gray dots indicated non-differential genes, red dots indicated high m6A methylation and high transcriptome expression, and blue dots indicated low m6A methylation and low transcriptome expression. The threshold range we set is |log2Fd| ≥ 1 (|fold change| ≥ 2). (**C**) KEGG pathway analysis results of DMGEs based on enrichment score (−log10(*p*-value)). (**D**) Protein–protein interaction network and hub gene identification of DMGEs. The 10 genes in the middle of the network diagram represented hub genes based on *p*-value using the MCODE plugin in Cytoscape as with the Hubba plugin, indicating a possible more dominant role in DMEGs. Darker colors represent stronger roles and lighter colors weaker roles.

To further demonstrate the association between m6A modification and gene mRNA expression, the intersection of the differentially expressed genes and differentially methylated m6A genes (DMEGs) was selected. By combining the mRNA-expression data and the m6A-methylation data in Venn diagrams, we identified 39 hyper-up DMEGs, and 3989 hypo-down DMEGs (Figure 6A). A scatter plot revealed m6A modification-associated gene expression profiles of 39 hyper-up DMEGs and 3989 hypo-down DMEGs between burn-injured skin and normal skin (Figure 6B). These results indicated that RNA m6A methylation may function in the regulation of mRNA expression levels after severe burn injury. 

Further enrichment analysis of KEGG showed that these DMEGs participated in endocytosis, lysosome, autophagy, the MAPK signaling pathway, and the EGFR tyrosine kinase inhibitor resistance pathway (Figure 6C). With 75 DMEGs enriched, the endocytosis pathway was essential for wound edge actin assembly and wound closure [30]. There are still 27 DMEGs associated with the adherens junctions pathway. It is believed that endocytic remodeling of adherens junctions is necessary for the establishment of “signaling centers” along wound margins that regulate actin assembly. Adherens junctions are a critical target for endocytosis during wound healing [31]. Insufficient autophagy in the epidermis prevents the production of dermal granulation tissue, wound healing, re-epithelialization, keratinocyte proliferation and differentiation, and mast cell and macrophage infiltration [32]. Activation of the MAPK signaling pathway can promote skin wound healing [33,34]. Epidermal growth factor (EGF) stimulates keratinocyte and fibroblast proliferation, allowing them to produce more angiogenic growth factors [35]. 

To find the hub genes of DMEGS which play critical roles mediated by m6A methylation, we analyzed the top 10 hub genes through the PPI data from STRING using the MCODE plugin in Cytoscape as with the Hubba plugin (Figure 6D). The top 10 hub genes were LOR, LCE3D, SPRR1A, LCE3E, LCE1B, CDSN, TGM1, EVPL, CASP14, and SPRR2G.

### 3.6. Identification of the Possible Molecular Functions of DMEGs after Burn Injury

To explore the possible molecular functions of DMEGs after burn injury, we first identified all statistically enriched terms, accumulative hypergeometric *p*-values, and enrichment factors that were calculated and used for filtering using the online tool Metascape [36].

**Figure 7:**
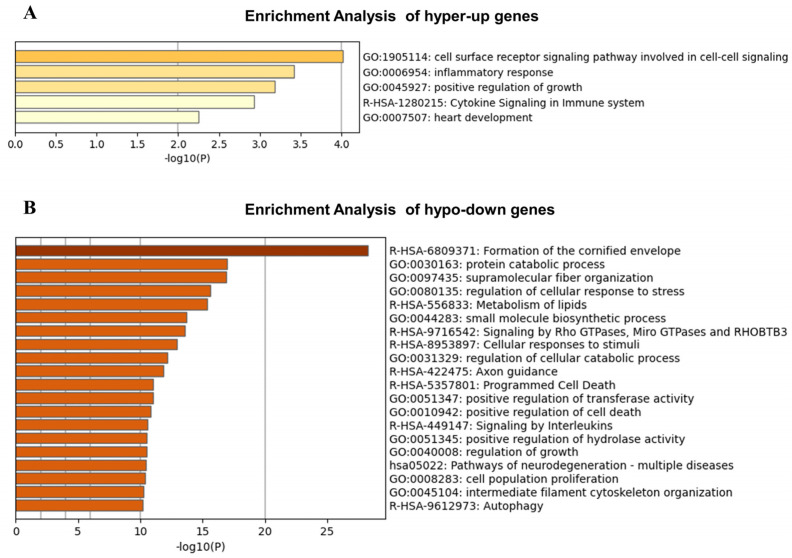
Enrichment analysis of DMEGs by calculating accumulative hypergeometric *p*-values and enrichment factors from all statistically enriched terms (can be GO/KEGG terms, canonical pathways, hallmark gene sets, etc.) on Metascape website (https://metascape.org, accessed on 1 June 2022). Bars were individually colored to represent rising *p*-values of statistical significance. (**A**) Enrichment analysis of hyper-methylated and up-expressed (hyper-up) genes. (**B**) Enrichment analysis of hypo-methylated and down-expressed(hypo-down) genes.

Enrichment analysis revealed that hypermethylated and upregulated (hyper-up) genes in the burn-injured skin and normal skin was involved in the cell surface receptor signaling pathway involved in cell–cell signaling (OPRM1, CSRP3, HTR3E), inflammatory response (CELA1, OPRM1, SLC11A1, CSRP3, HMOX2), positive regulation of growth (EIF4G1, FOXS1, SYT1, BMP4), and cytokine signaling in the immune system (EIF4G1, MMP1, OPRM1, SEM1) (Figure 7A). The top 20 significantly hypo-down genes based on foldchange are presented in Table 2. 

Enrichment analysis of hypomethylated and downregulated (hypo-down) genes identified the following downregulated pathways: formation of the cornified envelope, protein catabolic process, supramolecular fiber organization, regulation of cellular stress response, and metabolism of lipids (Figure 7B). The top 20 significantly hyper-up genes based on foldchange are presented in Table 3.

## 4. Discussion

Severe burns are the most traumatic and debilitating skin injuries, resulting in significant morbidity and mortality [30], which causes psychological and physical trauma to burn patients. In the early stages of burn injury, thermal stimulation causes skin trauma to appear with dysfunction of the skin barrier and persistent physiological derangements. The wound healing process is initiated after burn injury, which is a complex process dependent on the interaction of many cell types and mediators [37] and is highly complex in its spatial and temporal variability. 

This study extensively explored the alteration of gene expression including m6A regulators and potential m6A modification patterns in burn-injured and normal skin using human m6A-mRNA&lncRNA Epitranscriptomic microarray. We identified that many mRNAs and lncRNAs were hypomethylated in the burn-injured skin compared to normal skin. The methylation level of genes enriched in signaling pathways regulating the pluripotency of stem cells (ACVR1, BMP4, and NEUROG1), taste transduction (GNG13 and HTR3E), Morphine addiction (GNG13 and OPRM1) and the TGF-beta signaling pathway (ACVR1C and BMP4) were hypermethylated. The genes enriched in EGFR tyrosine kinase inhibitor resistance, endocytosis, the ErbB signaling pathway, and lysosome were hypomethylated. This study extends our knowledge of many lncRNAs related to wound healing to m6A methylation status. LncRNA Pvt1, LINC00302, and LncRNA MALAT1 are the most represented LncRNAs among the differently methylated lncRNAs, which have been found to function in sustaining stemness of epidermal progenitor cells [27], promoting the cell viability during burn injury repair [38], and targeting therapy for cutaneous wound healing and post-burn injury [39].

Moreover, we found that homeostasis of m6A in the skin was disrupted and there was a positive correlation between methylation and expression. m6A modifications in the skin may affect gene expression. Our results showed that 3989 hypomethylated mRNAs were down-expressed and inhibited many wound healing biological processes and pathways including the formation of the cornified envelope, protein catabolic process, supramolecular fiber organization, and regulation of cellular stress response; 39 hypermethylated mRNAs were up-expressed and enriched in the cell surface receptor signaling pathway, inflammatory response, cytokine signaling in the immune system, and positive regulation of growth. Among differentially m6A methylated and expressed genes, we discovered the top 10 hub genes (LOR, LCE3D, SPRR1A, LCE3E, LCE1B, CDSN, TGM1, EVPL, CASP14, and SPRR2G) which may play critical roles after burn injuries. Combined with the function of epigenetics, we have a good reason to presume that the function of m6A methylation modifications is mainly affected by severe burn injury, which induces inflammation, difficulty in wound healing, and susceptibility to infection. 

## 5. Conclusions

In summary, our study revealed for the first time that burn injury has an inhibitory effect on m6A modification and may inhibit wound repair by affecting m6A modification and expression of genes in wound healing-related pathways which provides new insight into wound healing after burn injury. Because of the complex stage- and state-specific roles of m6A modification, the m6A modification mechanism during the recovery of the burn-injured skin is yet to be revealed. Future studies based on these epigenetic clues could promote understanding of the potential molecular mechanism during the recovery of the burn-injured skin.

## Figures and Tables

**Table 1:** The primers for the quantitative real-time polymerase chain reaction.

Sequence		
Human Gene	Forward (5′-3′)	Reverse (5′-3′)
**METTL3**	ACTCTTGTAACCTATGCTGACCATTCC	TGACCTTCTTGCTCTGTTGTTCCTTAG
**METTL14**	ATTTTCCCACTGACCTTCCTCCTTTC	TGACCGTATTACATTCTGACTGCCATC
**WTAP**	GGGAAAGGACGGGGAGTGTT	AAGCATTCGACACTTCGCCA
**METTL16**	AATTGACATAGGCACGGGGG	TCTGTGGCACTTTCACCACT
**ALKBH5**	GACGTTCGAGCAATGGCAG	AGCAAGGCATGACTACCACC
**FTO**	GGTATCTCGCATCCTCATTGGTAATCC	ATGTCCACTTCATCTTGTCCGTTGTAG
**LRPPRC**	AGTTCAGTCTTCTGCTCGCC	AGGAACTGTGGACATGGCAG
**FMR1**	CATCAGTTGTAGCAGGGGAATC	ACGCAACTGGTCTACTTCCT
**HNRNPC**	AGACGAAGACTGAGCGGTTG	AGCCGAAAACAAGAAGGGGA
**GAPDH**	CTGGGCTACACTGAGCACC	AAGTGGTCGTTGAGGGCAATG

**Table 2:** The top 20 significantly hypo-down genes based on fold change.

Gene Symbol	Fold Change (Down-Expression)	*p*-Value (Expression)	Fold Change (Hypo-Methylation)	*p*-Value (m6A)	Regulation
SLURP1	111.7098	0.0009	29.7768	0.0029	Hypo-down
KRT2	67.6712	0.0054	47.3504	0.0029	Hypo-down
LCE2A	62.1089	0.0002	16.2295	0.0015	Hypo-down
HMGA1	58.5567	0.0018	39.1635	0.0073	Hypo-down
ATG7	50.4846	0.0139	44.2900	0.0294	Hypo-down
RARG	48.6102	0.0068	45.2373	0.0131	Hypo-down
LCE1C	46.8353	0.0015	13.5266	0.0019	Hypo-down
PFKP	45.7073	0.0045	50.4676	0.0067	Hypo-down
LCE2D	45.1279	0.0040	13.0420	0.0108	Hypo-down
NLRC5	43.9141	0.0094	45.3683	0.0096	Hypo-down
LCE5A	40.9822	0.0023	21.6845	0.0046	Hypo-down
MAML3	40.9162	0.0057	40.0430	0.0106	Hypo-down
KIRREL2	40.0545	0.0031	60.0066	0.0029	Hypo-down
RNASEH2C	39.2842	0.0029	30.4935	0.0083	Hypo-down
AHCTF1	38.4968	0.0032	36.8088	0.0077	Hypo-down
UNKL	36.7701	0.0104	42.2044	0.0213	Hypo-down
CD207	36.5578	0.0003	17.7942	0.0016	Hypo-down
SLC27A1	35.8739	0.0120	44.1601	0.0172	Hypo-down
FBXO18	35.1399	0.0237	33.4444	0.0344	Hypo-down
LCE6A	35.1080	0.0029	7.4851	0.0107	Hypo-down

**Table 3:** The top 20 significantly hyper-up genes based on fold change.

Gene Symbol	Fold Change (Up-Expression)	*p*-Value (Expression)	Fold Change (Hyper-m6A)	*p*-Value (m6A)	Regulation
MMP1	12.4473	0.0048	7.0151	0.0372	Hyper-up
CATG00000043842.1	7.3311	0.0081	7.1476	0.0224	Hyper-up
CATG00000091932.1	5.8154	0.0002	3.3018	0.0141	Hyper-up
SLC11A1	4.0354	0.0061	2.8741	0.0448	Hyper-up
OPRM1	3.9935	0.0044	4.6439	0.0134	Hyper-up
C21orf62	3.9124	0.0005	3.8339	0.0016	Hyper-up
CATG00000115972.1	3.9119	0.0002	3.4298	0.0006	Hyper-up
HTR3E	3.8389	0.0083	3.6604	0.0096	Hyper-up
CATG00000032515.1	3.7652	0.0042	4.4653	0.0183	Hyper-up
RANBP3L	3.7186	0.0100	3.2266	0.0121	Hyper-up
FOXS1	3.5274	0.0084	2.3571	0.0405	Hyper-up
SLC35G3	3.5218	0.0009	4.2904	0.0056	Hyper-up
SYT1	3.5067	0.0008	2.7735	0.0009	Hyper-up
C20orf96	3.4352	0.0006	3.3054	0.0019	Hyper-up
BMP4	3.3208	0.0157	3.1225	0.0469	Hyper-up
CATG00000034878.1	3.2484	0.0013	2.4300	0.0051	Hyper-up
CSRP3	3.1376	0.0053	2.8301	0.0142	Hyper-up
CATG00000049961.1	2.9691	0.0017	5.3561	0.0008	Hyper-up
HMOX2	2.9488	0.0105	2.9790	0.0318	Hyper-up
CELA1	2.9005	0.0181	3.6946	0.0204	Hyper-up

## Data Availability

Human m6A-mRNA Epitranscriptomic microarray data generated and analyzed for the present study have been deposited in the NCBI Gene Expression Omnibus data base (https://www.ncbi.nlm.nih.gov/geo/query/acc.cgi?acc=GSE214427, accessed on 30 September 2022).
